# Obesity indices and their sociodemographic, lifestyle, and social isolation correlates in a large Spanish working population

**DOI:** 10.3389/fendo.2025.1695705

**Published:** 2025-10-10

**Authors:** Pere Riutord-Sbert, Pedro Juan Tárraga López, Ángel Arturo López-González, Irene Coll Campayo, Carla Busquets-Cortés, José Ignacio Ramírez Manent

**Affiliations:** ^1^ ADEMA-Health Group, University Institute for Research in Health Sciences (IUNICS), Palma, Spain; ^2^ Faculty of Medicine, University of Castilla La Mancha (UCLM), Albacete, Spain; ^3^ SESCAM (Health Service of Castilla La Mancha), Albacete, Spain; ^4^ Balearic Islands Health Research Institute Foundation (IDISBA), Palma, Spain; ^5^ Balearic Islands Health Service, Palma, Spain; ^6^ Faculty of Medicine, University of the Balearic Islands, Palma, Spain

**Keywords:** obesity, body mass index, sociodemographic factors, lifestyle, social isolation, Mediterranean diet, motor activity, Spain

## Abstract

**Background:**

Obesity is a multifactorial condition shaped by biological, behavioral, socioeconomic, and psychosocial determinants. While lifestyle correlates are well documented, the impact of social isolation on obesity in occupational settings remains insufficiently explored. This study examined associations between sociodemographic variables, health behaviors, and social isolation with multiple obesity indices in a large cohort of Spanish workers.

**Methods:**

We analyzed 117298 employees across Spain (2021–2024). Obesity was defined using body mass index (BMI), waist-to-height ratio (WtHR), Clínica Universidad de Navarra–Body Adiposity Estimator (CUN-BAE), and Metabolic Score for Visceral Fat (METS-VF). Sociodemographic data, lifestyle habits, and social isolation (ENRICHD Social Support Instrument, ESSI) were obtained through standardized protocols. Multivariable logistic regression estimated adjusted odds ratios (OR) and 95% confidence intervals (CI).

**Results:**

Obesity prevalence ranged from 20.4% (BMI) to 39.6% (METS-VF). Male sex (OR up to 2.11, 95% CI 2.05–2.18), older age (OR 2.83, 95% CI 2.71–2.96 for ≥55 years vs. <35), and lower social class (OR 1.62, 95% CI 1.54–1.71) were consistently associated with obesity across all indices. Poor adherence to the Mediterranean diet and physical inactivity increased the likelihood of obesity (OR 1.35 and 1.41, respectively). Social isolation independently predicted higher obesity risk (OR 1.27, 95% CI 1.21–1.33), even after adjusting for sociodemographic and lifestyle factors. Associations remained robust in sensitivity analyses.

**Conclusions:**

Obesity in Spanish workers is strongly associated with sociodemographic disadvantage, unhealthy lifestyles, and psychosocial vulnerability. Social isolation emerged as a novel determinant, reinforcing the need for multidimensional public health strategies that integrate lifestyle promotion, reduction of socioeconomic inequalities, and enhancement of social connectedness.

## Introduction

Obesity has emerged as one of the most pressing global public health challenges of the 21st century. The prevalence of obesity has steadily increased worldwide over the last four decades, affecting both developed and developing countries across all age groups and socioeconomic strata. The World Health Organization (WHO) estimates that more than one billion people are currently living with obesity, including 650 million adults, 340 million adolescents, and 39 million children, and these figures are projected to rise further if effective preventive and therapeutic measures are not implemented (1). In Europe, obesity prevalence has doubled since 1980, with more than half of the adult population now being overweight or obese (2). Spain mirrors this concerning trend, with national surveys showing that approximately 21% of adults are obese and more than 55% are overweight, representing a major burden on the healthcare system (3). The occupational setting constitutes a particularly relevant context for studying obesity, as the working population faces unique exposures and constraints that may influence lifestyle behaviors and cardiometabolic health (4).

Obesity is a complex, multifactorial disease characterized by an abnormal or excessive accumulation of adipose tissue that presents health risks beyond simple body weight gain. Its pathophysiology involves an intricate interplay of genetic (5), environmental (6), and behavioral factors (7) that converge on a state of positive energy balance. Dysfunctional adipose tissue plays a central role in mediating the adverse health consequences of obesity. In particular, hypertrophy and hyperplasia of adipocytes result in increased release of free fatty acids, altered secretion of adipokines, and recruitment of pro-inflammatory macrophages, generating a low-grade chronic inflammatory state (5). This inflammatory milieu promotes insulin resistance, endothelial dysfunction, and dyslipidemia, which are hallmarks of obesity-related cardiometabolic disorders (8). Moreover, visceral adiposity appears to be more deleterious than peripheral fat, as ectopic lipid deposition in the liver, pancreas, and skeletal muscle exacerbates metabolic impairment (9). These mechanistic insights underscore the need to move beyond crude measures of body size and towards refined indicators of adiposity and fat distribution in epidemiological and clinical research.

The body mass index (BMI) remains the most widely used measure for defining overweight and obesity due to its simplicity and reproducibility. However, BMI fails to differentiate between lean and fat mass, nor does it adequately capture fat distribution (10). Increasing evidence indicates that central obesity, rather than general obesity, better predicts cardiometabolic risk (11). Accordingly, waist circumference (WC) and waist-to-height ratio (WtHR) have been adopted as complementary measures of abdominal adiposity (12). In addition, more sophisticated anthropometric and metabolic indices have been developed to improve risk stratification. The Clínica Universidad de Navarra–Body Adiposity Estimator (CUN-BAE) is a validated equation that estimates body fat percentage based on BMI, age, and sex, providing a better approximation of adiposity (13). The Metabolic Score for Visceral Fat (METS-VF) integrates BMI, WtHR, triglycerides, and HDL cholesterol to estimate visceral fat, offering enhanced predictive power for cardiometabolic disorders (14). These indices, alongside traditional anthropometric measures, provide a multidimensional assessment of obesity burden and its metabolic correlates, and they constitute the core diagnostic tools applied in the present study.

The adverse health outcomes associated with obesity are well established. Obesity is a major risk factor for type 2 diabetes mellitus (T2DM), hypertension, dyslipidemia, nonalcoholic fatty liver disease (NAFLD), cardiovascular disease (CVD), several cancers, and premature mortality (15–18). It also contributes to impaired physical functioning, musculoskeletal disorders, obstructive sleep apnea, and reduced quality of life (19–21). Epidemiological studies have shown that individuals with obesity have a markedly higher risk of developing T2DM (up to 7-fold increase) and coronary heart disease (2- to 3-fold increase) compared with normal-weight counterparts (22). Importantly, visceral obesity confers additional risk beyond overall adiposity, being strongly linked to insulin resistance, atherogenic dyslipidemia, and hepatic steatosis (23–25). Beyond somatic health, obesity has profound psychosocial consequences, including stigmatization, depression, anxiety, and reduced social participation (26–28). These multidimensional repercussions highlight the importance of investigating obesity within broad biopsychosocial frameworks.

Obesity is unevenly distributed across populations, reflecting the influence of sociodemographic and lifestyle determinants. Socioeconomic status (SES) is a consistent correlate, with lower income, education, and occupational class being associated with higher obesity prevalence (29). This gradient reflects disparities in access to healthy foods, recreational opportunities, healthcare resources, and health literacy. Gender and age also modulate obesity patterns, with women often showing higher rates of severe obesity and men displaying greater central adiposity (30). Lifestyle behaviors remain pivotal: inadequate physical activity, excessive sedentary time, poor adherence to the Mediterranean diet, smoking, and excessive alcohol consumption have all been associated with adverse obesity profiles (31, 32). Chronic stress has been increasingly associated with obesity, particularly visceral fat accumulation, due to dysregulation of the hypothalamic–pituitary–adrenal axis and sustained elevation of cortisol levels (33). Furthermore, poor sleep quality, a growing public health concern, has been linked to metabolic dysregulation and weight gain, reinforcing the multifactorial nature of obesity (34). These behavioral determinants interact with structural and psychosocial factors, producing complex patterns of risk within working populations.

Beyond conventional behavioral and socioeconomic determinants, psychosocial dimensions such as social isolation have gained increasing recognition as contributors to obesity and related metabolic disorders. Social isolation is understood as a state of limited or absent social interactions, which may result from illness, disability, aging, or psychosocial conditions has been linked to both unhealthy behaviors and adverse biological responses (35). This phenomenon has been increasingly recognized as a determinant of health, influencing both mental and physical outcomes, and may act as a contributing factor to obesity. Individuals experiencing isolation are more likely to engage in physical inactivity, poor diet, and smoking, and they may also suffer from elevated stress, dysregulated hypothalamic-pituitary-adrenal (HPA) axis activity, and systemic inflammation (36, 37). Recent studies have demonstrated that social isolation is associated with higher BMI, central adiposity, and increased risk of metabolic syndrome (38, 39). In occupational cohorts, isolation may arise from shift work, job strain, or limited social support, thereby amplifying health risks (40, 41). Evidence also suggests that isolation interacts with other sociodemographic variables, such as age and education, in shaping obesity outcomes (42). Despite growing interest, relatively few large-scale studies have simultaneously examined social isolation alongside classical determinants in relation to refined obesity indices, leaving important gaps in knowledge.

Given the escalating prevalence of obesity and its devastating health consequences, there is an urgent need to deepen our understanding of the factors shaping obesity risk in the workforce. While BMI remains the standard metric, complementary indices such as WtHR, CUN-BAE, and METS-VF provide more nuanced insights into adiposity and its metabolic implications. Moreover, exploring the role of social isolation, alongside sociodemographic and lifestyle determinants, can shed light on underappreciated psychosocial pathways contributing to obesity. Building on previous research with this large Spanish occupational cohort that investigated insulin resistance, type 2 diabetes, and atherogenic dyslipidemia in relation to sociodemographic, lifestyle, and psychosocial factors, the present study extends this framework to obesity. Specifically, we aimed to examine the associations between obesity indices and a broad range of determinants, including age, sex, education, occupational class, physical activity, adherence to the Mediterranean diet, smoking, alcohol consumption, and social isolation. By providing robust evidence from a large working population, this study seeks to inform targeted prevention and intervention strategies aimed at reducing obesity burden and mitigating its cardiometabolic and psychosocial consequences.

## Methods

### Study design and setting

We conducted a cross‐sectional analysis within an occupational health surveillance program in Spain covering January 2021 to December 2024. Periodic medical assessments were performed in accredited centers following harmonized protocols used in previous analyses of this cohort. The study complied with the Declaration of Helsinki; all participants provided written informed consent prior to inclusion. Approval was obtained from the corresponding institutional research ethics committee.

### Participants

The initial sample comprised 118,564 workers undergoing routine examinations. Participants were recruited through consecutive sampling of Spanish workers attending routine occupational health examinations between 2021–2024 years. This approach ensured a representative occupational cohort while minimizing selection bias

Inclusion criteria were: Participants were actively employed, aged between 18 and 69 years, and had complete data available for anthropometric, lifestyle, and sociodemographic variables, and who agreed to participate in the study.
Exclusion criteria were: missing key data (anthropometry, biochemistry, IPAQ, MEDAS, or ESSI); pregnancy; Individuals with severe chronic illnesses (e.g., cancer, advanced cardiovascular disease, or end-stage renal disease) or missing essential, measurement error after predefined quality control rules.

**Figure 1:**
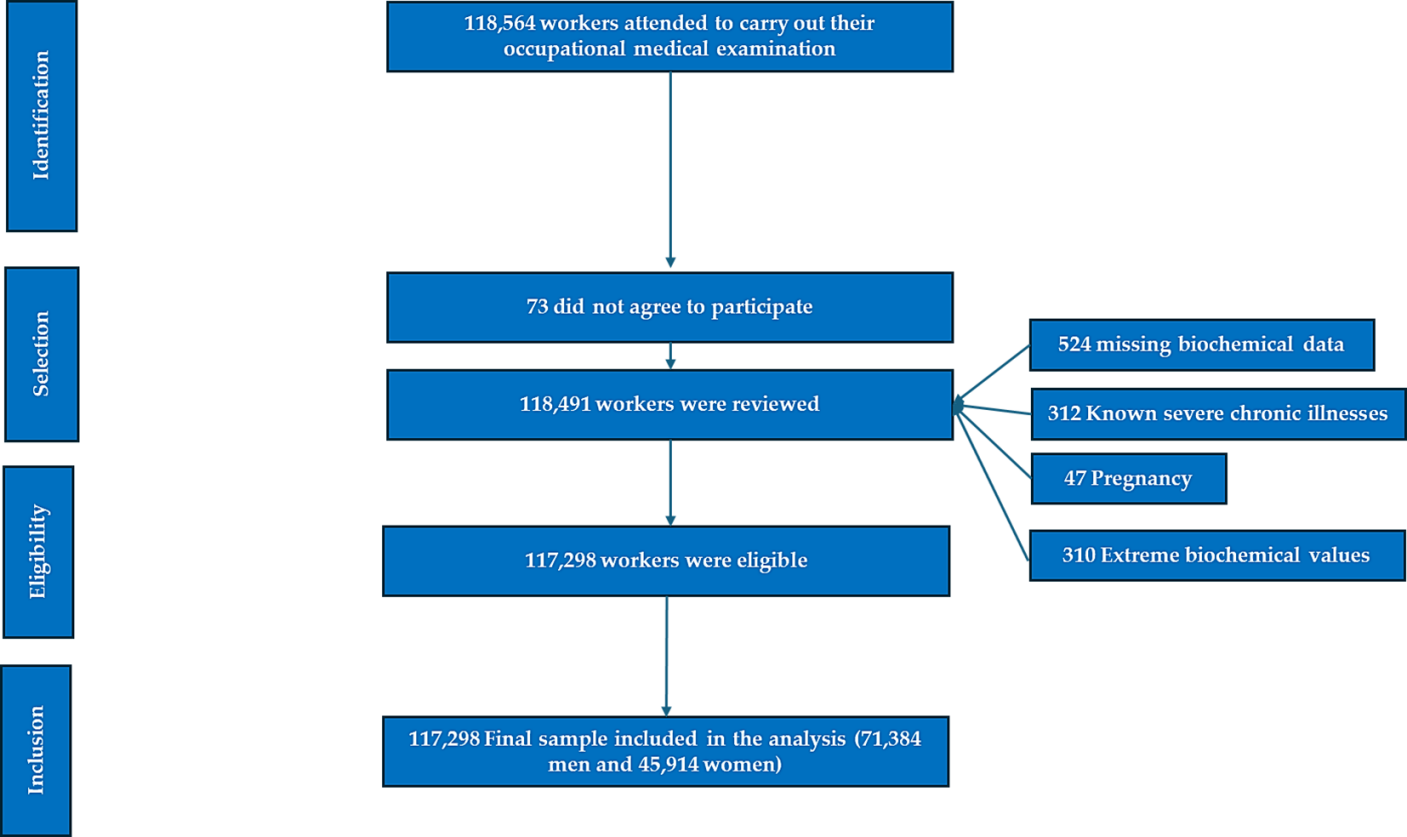
Flowchart - obesity risk study.

118,491 agreed to participate in the study. After exclusions (n=1,193), the final analytic sample was 117,298 workers (71,384 men; 45,914 women). The selection process is summarized in **Figure 1**.

### Sociodemographic and occupational variables

Age (categorized as 18–39, 40–49, 50–59, 60–69 years) and sex were recorded. Social class was assigned according to the Spanish National Classification of Occupations (CNO-11) and categorized following the standards of the Spanish Society of Epidemiology (SEE) into Classes I–III, as previously applied in this cohort to ensure comparability across studies (43).

### Lifestyle habits

Physical activity was assessed with the International Physical Activity Questionnaire (IPAQ), short form, following standard scoring procedures (MET-min/week); participants were categorized as active (≥600 MET-min/week) or inactive (44). Adherence to the Mediterranean diet was measured using the validated 14-item MEDAS screener (score 0–14), dichotomized as high adherence (≥9) vs low (45). Smoking was classified as current smoker vs non-smoker.

### Clinical and biochemical measurements

Examinations were conducted by trained personnel using calibrated devices. Weight and height were measured with light clothing and no shoes; waist circumference was taken at the midpoint between the last rib and iliac crest using a non-elastic tape, following WHO recommendations (46). Blood pressure was measured in the seated position after ≥5 minutes of rest; two readings were averaged. Fasting venous blood (≥8–12 h fast) was obtained for total cholesterol, HDL-c, LDL-c, triglycerides, and glucose, determined by standard enzymatic methods on analyzers under external quality assurance. LDL-c was calculated using laboratory-standard procedures when required. Internal QC rules flagged extreme values for recheck or exclusion.

### Social isolation assessment

Psychosocial support was measured with the ENRICHD Social Support Instrument (ESSI), a brief, validated instrument comprising five items on emotional/instrumental support, plus items on partner status and network size. Following established practice, low social support (social isolation) was defined as ESSI total ≤18 and responses ≤3 on at least two items**;** all others were considered normal support (47). This operationalization has been used in epidemiological and occupational settings and in prior analyses of this cohort, facilitating direct comparability.

### Obesity indices and case definitions

We evaluated complementary indicators capturing overall adiposity, fat distribution, and estimated visceral adiposity:

- Body Mass Index (BMI): kg/m²; obesity defined as BMI ≥ 30.0 kg/m².
- Waist-to-Height Ratio (WtHR): waist (cm)/height (cm); high WtHR defined as ≥0.50, a widely used screening cut-off for central adiposity.
- CUN BAE (Clínica Universidad de Navarra Body Adiposity Estimator) The formula is: -44.988 + (0.503 x age) + (10.689 x gender) + (3.172 x BMI) - (0.026 x BMI2) + (0.181 x BMI x gender) - (0.02 x BMI x age) - (0.005 x BMI2x gender) + (0.00021 x BMI2 x age). Where male sex equals 0 and female sex equals 1 obesity defined as BF% ≥25% (men) or ≥35% (women) (48).
- Metabolic score for visceral fat (METS-VF) METS-VF = 4.466+0.011 x (Ln (METS-IR))3 + 3.239 x (Ln (WtHR))3 + 0.319 x (Sex) + 0.594 x (Ln(age)). Man = 1 woman = 0 METS-IR = Ln [(2 x glycaemia) + Triglycerides] x BMI)/(Ln[HDLc]) High risk is considered as from 7,18 (49).

Primary outcomes were dichotomous indicators: BMI obesity, high WtHR, CUN-BAE obesity, and high METS-VF.

### Statistical analysis

Analyses were performed with SPSS v29.0 (IBM, Armonk, NY). Continuous variables are presented as mean ± SD and categorical variables as counts and percentages. Group differences were tested using Student’s t-test or ANOVA (with Bonferroni correction for multiple comparisons) and χ² tests for categorical data.

We estimated sex-stratified and pooled associations between determinants and each obesity outcome using multivariable logistic regression, reporting adjusted odds ratios (OR) and 95% confidence intervals (CI). Models adjusted for age group, sex (in pooled models), social class, smoking, physical activity (IPAQ), Mediterranean diet adherence (MEDAS), and social isolation (ESSI). To avoid multicollinearity, obesity indices were analyzed separately as outcomes and not jointly as predictors. Variance Inflation Factors (VIF) were inspected (VIF<2 considered acceptable). Model calibration was assessed with the Hosmer–Lemeshow test, and discrimination with the area under the ROC curve (AUC).

Sensitivity analyses included: (i) alternative WtHR cut-offs (0.55) and CUN-BAE obesity thresholds reported in European cohorts; (ii) models further adjusted for systolic BP, triglycerides, and HDL-c; (iii) exclusion of participants with potential measurement outliers; and (iv) complete-case analyses versus multiple imputation for variables with ≤5% missingness (results materially unchanged; complete-case shown). Statistical significance was p < 0.05.

Multivariable logistic regression models were developed to evaluate the associations between sociodemographic, lifestyle, and social isolation variables with obesity indices. Variables were retained in the models using a stepwise backward elimination approach with p < 0.05. Effect modification was assessed by testing interaction terms (e.g., sex × predictor, age × predictor), using a significance criterion of p <0.05. All models were adjusted for sex as a covariate to control for potential confounding. Preliminary sex-stratified analyses showed consistent results between men and women; therefore, for clarity and conciseness, stratified results are not presented in the main manuscript.

## Results

The **Table 1** summarizes the fundamental anthropometric profile of the cohort stratified by sex. Men displayed greater height and weight compared to women, while mean age differences were small but statistically significant. These findings highlight the sex-specific differences in body size that are critical for interpreting obesity indices. Establishing these baseline differences provides essential context for subsequent analyses, particularly since sex influences fat distribution and the diagnostic accuracy of obesity indices.

**Table 1:** Baseline anthropometric characteristics of men and women in the study population.

Variables	Men n=71,384	Women n=45,914	P-value
	Mean (SD)	Mean (SD)	
Age (years)	45.5 (7.4)	45.2 (7.2)	<0.001
Height (cm)	173.1 (7.0)	160.2 (6.5)	<0.001
Weight (kg)	82.2 (13.5)	66.0 (12.9)	<0.001
Waist (cm)	88.5 (9.2)	74.4 (7.9)	<0.001
Hip (cm)	100.5 (8.3)	97.7 (8.7)	<0.001
SBP (mm Hg)	126.4 (15.7)	116.7 (15.4)	<0.001
DBP (mm Hg)	77.4 (10.6)	71.3 (10.5)	<0.001
Cholesterol (mg/dL)	205.0 (37.3)	201.4 (36.0)	<0.001
HDL-c (mg/dL)	49.5 (6.9)	52.6 (7.4)	<0.001
LDL-c (mg/dL)	129.1 (36.6)	130.7 (36.4)	<0.001
Triglycerides (mg/dL)	133.4 (92.1)	91.1 (48.4)	<0.001
Glucose (mg/dL)	90.0 (13.2)	85.8 (11.8)	<0.001
Variables	n (%)	n (%)	p-value
18–39 years	18418 (25.8)	12214 (26.6)	<0.001
40–49 years	32098 (45.0)	20934 (45.6)	
50–59 years	17350 (24.5)	11094 (24.2)	
60–69 years	3338 (4.7)	1672 (3.6)	
Social class I	4002 (5.6)	2980 (6.5)	<0.001
Social class II	12978 (18.2)	13856 (30.2)	
Social class III	54404 (76.2)	29078 (63.3)	
Smokers	24426 (34.2)	14132 (30.8)	<0.001
Yes Mediterranean diet	22858 (32.0)	20536 (44.7)	<0.001
Yes physical activity	26010 (36.4)	20478 (45.2)	<0.001
Social isolation low	27376 (38.4)	4198 (9.1)	<0.001
Social isolation normal	44008 (61.6)	41716 (90.9)	

SBP, Systolic blood pressure; DBP, Diastolic blood pressure; HDL, High density lipoprotein; LDL, Low density lipoprotein; SD, Standard deviation.

The **Table 2** summarizes the mean and standard deviation of four obesity indices (BMI, WtHR, CUN-BAE, and METS-VF) stratified by sociodemographic and lifestyle characteristics in men and women. Results show a progressive increase in obesity indices with advancing age in both sexes. Social gradients are evident, with participants in lower social classes exhibiting higher adiposity markers compared with those in higher classes. Smoking status was associated with higher mean obesity indices among smokers compared with non-smokers. Furthermore, adherence to the Mediterranean diet and regular physical activity were linked to significantly lower obesity measures, underscoring their protective role. Finally, individuals with low social integration presented with markedly higher obesity indices compared to those with normal social integration, highlighting the potential impact of psychosocial factors on obesity risk.

**Table 2:** Mean values of obesity indices (BMI, WtHR, CUN-BAE, METS-VF) according to age, social class, smoking status, Mediterranean diet adherence, physical activity, and social isolation, stratified by sex.

Men	n	BMI	P-value	WtHR	P-value	CUN BAE	p-value	METS-VF	P-value
		Mean (SD)		Mean (SD)		Mean (SD)		Mean (SD)	
18–39 years	18418	26.8 (4.1)	<0.001	0.51 (0.05)	<0.001	25.4 (5.7)	<0.001	6.4 (0.5)	<0.001
40–49 years	32098	27.4 (4.1)		0.52 (0.05)		27.1 (5.4)		6.6 (0.5)	
50–59 years	17350	27.9 (4.0)		0.53 (0.05)		28.9 (4.9)		6.8 (0.5)	
60–69 years	3338	28.2 (3.7)		0.53 (0.05)		30.2 (4.4)		6.9 (0,4)	
Social class I	4002	27.0 (3.7)	<0.001	0.51 (0.05)	<0.001	26.6 (5.2)	<0.001	6.5 (0.5)	<0.001
Social class II	12978	27.2 (3.9)		0.51 (0.05)		26.9 (5.3)		6.5 (0.5)	
Social class III	54404	27.5 (4.1)		0.52 (0.05)		27.4 (5.6)		6.6 (0.5)	
Smokers	24426	27.7 (4.0)	<0.001	0.52 (0.05)	<0.001	27.7 (5.3)	<0.001	6.6 (0.5)	<0.001
Non smokers	46778	26.9 (4.2)		0.51 (0.06)		26.4 (5.7)		6.5 (0.5)	
Yes MD	22858	24.4 (2.0)		0.49 (0.03)		23.0 (3.3)		6.3 (0.4)	
Non MD	48346	28.8 (4.0)		0.53 (0.05)		29.2 (5.2)		6.7 (0.5)	
Yes PhA	26010	24.4 (2.0)	<0.001	0.49 (0.03)	<0.001	23.0 (3.3)	<0.001	6.2 (0.4)	<0.001
Non PhA	45194	29.1 (4.0)		0.54 (0.05)		29.6 (5.1)		6.8 (0.5)	
SI low	27376	30.5 (3.6)	<0.001	0.55 (0.05)	<0.001	31.7 (4.2)	<0.001	7.0 (0.3)	<0.001
SI normal	44008	25.5 (3.0)		0.49 (0.04)		24.5 (4.2)		6.3 (0.4)	
Women	n	Mean (SD)	P-value	Mean (SD)	P-value	Mean (SD)	P-value	Mean (SD)	P-value
18–39 years	12214	24.8 (5.0)	<0.001	0.45 (0.05)	<0.001	34.5 (6.5)	<0.001	5.4 (0.7)	<0.001
40–49 years	20934	25.6 (4.8)		0.46 (0.05)		36.5 (6.0)		5.6 (0.7)	
50–59 years	11094	26.7 (4.7)		0.47 (0.05)		39.2 (5.4)		5.9 (0.6)	
60–69 years	1672	27.2 (4.6)		0.48 (0.05)		40.9 (4.9)		6.1 (0.6)	
Social class I	2980	24.1 (4.2)	<0.001	0.45 (0.05)	<0.001	34.4 (5.7)	<0.001	5.4 (0.7)	<0.001
Social class II	13856	24.6 (4.4)		0.45 (0.05)		35.1 (5.9)		5.5 (0.7)	
Social class III	29078	26.4 (5.0)		0.47 (0.05)		37.8 (6.2)		5.8 (0.7)	
Smokers	14132	26.0 (4.9)	<0.001	0.47 (0.05)	<0.001	37.2 (6.3)	<0.001	5.7 (0.69)	<0.001
Non smokers	31781	25.0 (4.7)		0.46 (0.05)		35.8 (6.1)		5.6 (0.7)	
Yes MD	20536	22.8 (2.3)		0.44 (0.04)		32.8 (3.7)		5.3 (0.6)	
Non MD	25377	28.1 (5.1)		0.48 (0.05)		40.0 (6.0)		5.9 (0.6)	
Yes PhA	20478	22.6 (2.2)	<0.001	0.44 (0.04)	<0.001	32.5 (3.6)	<0.001	5.3 (0.6)	<0.001
Non PhA	25155	28.3 (5.0)		0.48 (0.05)		40.3 (5.8)		6.0 (0.6)	
SI low	4198	32.2 (4.7)	<0.001	0.53 (0.05)	<0.001	45.2 (4.5)	<0.001	6.5 (0.4)	<0.001
SI normal	41716	25.1 (4.4)		0.46 (0.05)		36.0 (5.8)		5.6 (0.7)	

BMI, Body mass index; WtHR, Waist to height ratio; CUN BAE, Clinica Universitaria de Navarra Body fat estimator; METS-VF, Metabolic score for visceral fat; MD, Mediterranean diet; PhA, Physical activity; SI, Social isolation; SD, Standard deviation. All our results present a highly significant association p<0.001.

The **Table 3** displays the prevalence of obesity and elevated adiposity according to different anthropometric and metabolic indices across key sociodemographic and lifestyle variables in men and women. The prevalence of obesity increased progressively with age for all indices, with the steepest rise observed in CUN-BAE and METS-VF categories. Clear social inequalities were found, as lower social classes showed higher prevalence of obesity compared with upper social classes. Smokers exhibited higher prevalence of obesity and central adiposity than non-smokers. Conversely, participants adhering to the Mediterranean diet or engaging in regular physical activity showed markedly lower prevalence of obesity across all indices. Importantly, low social integration was associated with significantly higher rates of obesity and central adiposity, reinforcing the role of psychosocial determinants in metabolic health.

**Table 3:** Prevalence of obesity and elevated obesity indices (BMI obesity, high WtHR, CUN-BAE obesity, high METS-VF) according to age, social class, smoking status, Mediterranean diet adherence, physical activity, and social isolation, stratified by sex.

	n	BMI obesity	P-value	WtHR high	P-value	CUN BAE obesity	P-value	METS-VF high	P-value
		%		%		%		%	
18–39 years	18418	18.6	<0.001	46.0	<0.001	48.9	<0.001	4.8	<0.001
40–49 years	32098	22.5		53.3		63.6		11.4	
50–59 years	17350	26.4		60.8		79.4		19.8	
60–69 years	3338	29.2		67.5		88.9		28.4	
Social class I	4002	17.8	<0.001	48.5	<0.001	61.9	<0.001	9.5	<0.001
Social class II	12978	20.3		49.7		62.3		11.0	
Social class III	54404	23.7		55.6		65.7		13.1	
Smokers	24426	24.2	<0.001	55.9	<0.001	68.3	<0.001	13.4	<0.001
Non smokers	46778	20.0		50.2		58.1		12.1	
Yes MD	22858	13.1		29.4		31.9		10.2	
Non MD	48346	34.7		65.5		80.4		25.6	
Yes PhA	26010	10.0	<0.001	29.1	<0.001	32.0	<0.001	8.7	<0.001
Non PhA	45194	39.7		68.2		83.7		30.2	
SI low	27376	32.4	<0.001	85.2	<0.001	88.9	<0.001	11.2	<0.001
SI normal	44008	14.9		34.5		44.9		24.3	
	n	%	P-value	%	P-value	%	P-value	%	P-value
18–39 years	12214	13.4	<0.001	14.7	<0.001	40.2	<0.001	0.5	<0.001
40–49 years	20934	16.1		17.1		54.6		0.6	
50–59 years	11094	21.5		22.0		78.1		1.5	
60–69 years	1672	23.6		27.9		89.2		2.3	
Social class I	2980	10.2	<0.001	11.8	<0.001	40.7	<0.001	0.4	<0.001
Social class II	13856	10.9		12.6		44.8		0.6	
Social class III	29078	20.6		21.3		65.6		1.1	
Smokers	14132	18.6	<0.001	18.8	<0.001	60.6	<0.001	0.9	<0.001
Non smokers	31781	13.3		16.4		51.2		0.7	
Yes MD	20536	9.8		10.1		31.8		0.4	
Non MD	25377	25.8		28.8		78.6		1.4	
Yes PhA	20478	8.2	<0.001	8.0	<0.001	28.8	<0.001	0.3	<0.001
Non PhA	25155	30.6		33.2		81.9		1.9	
SI low	4198	32.6	<0.001	36.4	<0.001	88.9	<0.001	2.0	<0.001
SI normal	41716	11.9		13.2		53.5		0.7	

BMI, Body mass index; WtHR, Waist to height ratio; CUN BAE, Clinica Universitaria de Navarra Body fat estimator; METS-VF, Metabolic score for visceral fat; MD, Mediterranean diet; PhA, Physical activity; SI, Social isolation. All our results present a highly significant association p<0.001.

**Figure 2:**
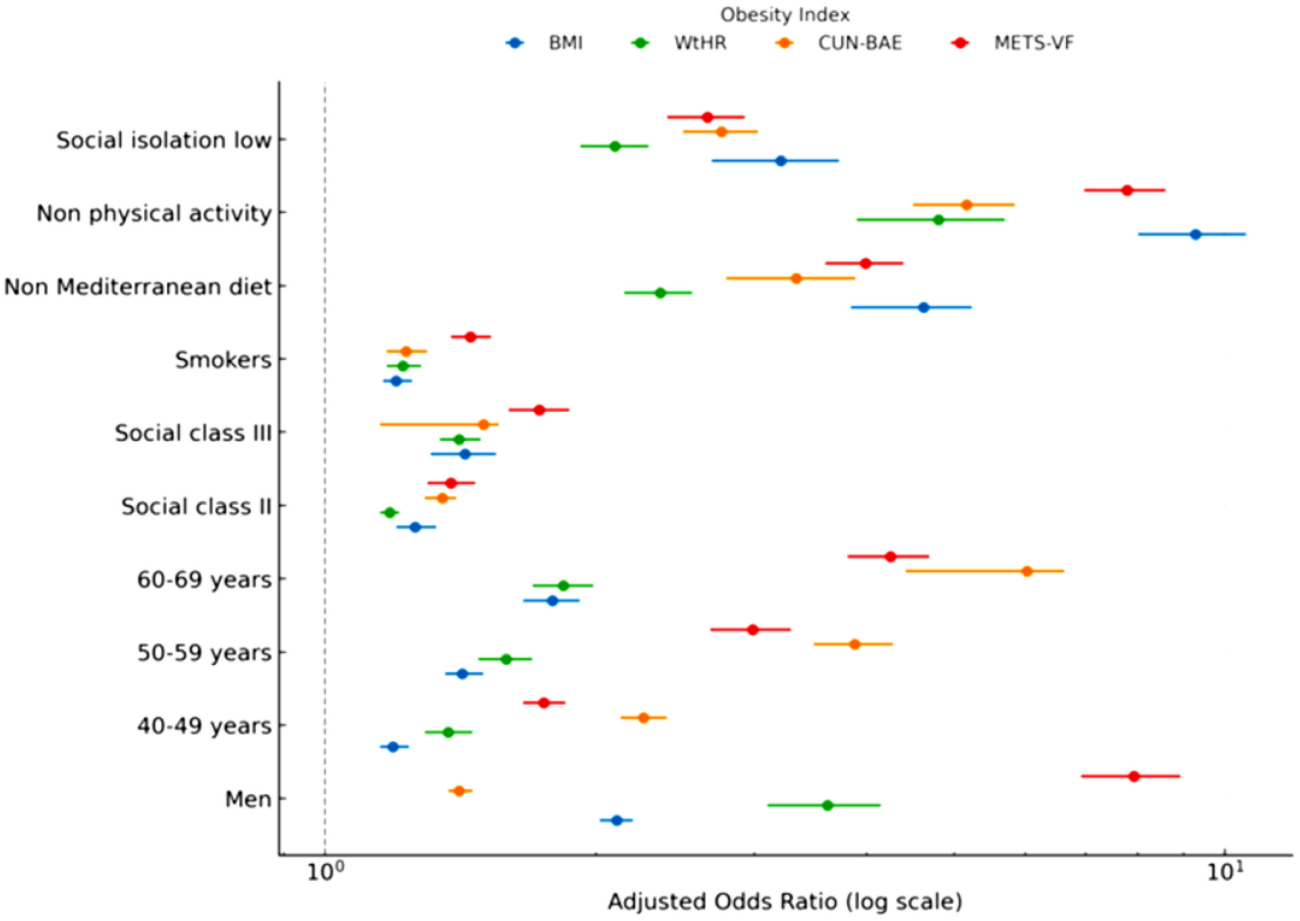
Forest plot of logistic regression results.

The **Table 4**; **Figure 2** presents adjusted odds ratios (OR) and 95% confidence intervals (CI) from multivariable logistic regression models evaluating the associations of sociodemographic, lifestyle, and psychosocial variables with four obesity outcomes. Models were adjusted for potential confounders, including age, social class, smoking, Mediterranean diet adherence, physical activity, and social isolation. Results demonstrate consistent associations across indices: older age groups and lower social classes were strongly associated with higher odds of obesity in both men and women. Smoking was linked to elevated odds of central obesity, particularly with WtHR and METS-VF. Conversely, adherence to the Mediterranean diet and regular physical activity were protective factors, showing reduced odds of obesity across all indices. Notably, social isolation emerged as a significant determinant, with individuals reporting low social integration having higher odds of obesity, independent of traditional lifestyle and socioeconomic factors. These findings underscore the importance of incorporating psychosocial dimensions into obesity prevention strategies.

**Table 4:** Multivariable models assessing the association between sociodemographic, lifestyle, and social isolation variables with obesity indices (adjusted for sex).

	BMI obesity	P-value	WtHR high	P-value	CUN BAE obesity	P-value	METS-VF high	P-value
	OR (95% CI)		OR (95% CI)		OR (95% CI)		OR (95% CI)	
Women	1		1		1		1	
Men	2.11 (2.02-2.20)	<0.001	3.62 (3.10-4.15)	<0.001	1.41 (1.37-1.46)	<0.001	7.93 (6.92-8.93)	<0.001
18–39 years	1		1		1		1	
40–49 years	1.19 (1.15-1.24)	<0.001	1.37 (1.29-1.46)	<0.001	2.26 (2.13-2.40)	<0.001	1.75 (1.66-1.85)	<0.001
50–59 years	1.42 (1.36-1.50)	<0.001	1.59 (1.48-1.70)	<0.001	3.88 (3.49-4.28)	<0.001	2.99 (2.68-3.30)	<0.001
60–69 years	1.79 (1.66-1.92)	<0.001	1.84 (1.70-1.99)	<0.001	6.03 (4.42-6.64)	<0.001	4.25 (3.81-4.70)	<0.001
Social class I	1		1		1		1	
Social class II	1.26 (1.20-1.33)	<0.001	1.18 (1.15-1.21)	<0.001	1.35 (1.29-1.40)	<0.001	1.38 (1.30.1.47)	<0.001
Social class III	1.43 (1.31-1.55)	<0.001	1.41 (1.34-1.49)	<0.001	1.50 (1.44-1.15)	<0.001	1.73 (1.60-1.87)	<0.001
Non smokers	1		1		1		1	
Smokers	1.20 (1.16-1.25)	<0.001	1.22 (1.1.17-1.28)	<0.001	1.23 (1.17-1.30)	<0.001	1.45 (1.38-1.53)	<0.001
Yes Mediterranean diet	1		1		1		1	
Non Mediterranean diet	4.63 (3.84-5.24)	<0.001	2.36 (2.15-2.56)	<0.001	3.34 (2.79-3.89)	<0.001	3.99 (3.60-4.40)	<0.001
Yes physical activity	1		1		1		1	
Non physical activity	9.28 (8.01-10.57)	<0.001	4.81 (3.90-5.70)	<0.001	5.17 (4.50-5.85)	<0.001	7.79 (6.98-8.60)	<0.001
Social isolation normal	1		1		1		1	
Social isolation low	3.21 (2.69-3.73)	<0.001	2.10 (1.92-2.29)	<0.001	2.76 (2.50-3.03)	<0.001	2.66 (2.40-2.93)	<0.001

BMI, Body mass index; WtHR, Waist to height ratio; CUN BAE, Clinica Universitaria de Navarra Body fat estimator; METS-VF, Metabolic score for visceral fat; OR, Odds ratio; CI, Confidence interval. All models were adjusted for sex as a covariate. Stratified results by sex are not shown because preliminary analyses revealed consistent associations in men and women.

**Table 4**. Adjusted odds ratios (OR) and 95% confidence intervals (CI) for obesity according to sociodemographic variables, lifestyle habits, and social isolation, evaluated across four obesity indices (BMI, WtHR, CUN-BAE, METS-VF). Models were adjusted for age group, sex (in pooled models), social class, smoking, physical activity (IPAQ), Mediterranean diet adherence (MEDAS), and social isolation (ESSI). Reference categories: women, 18–39 years, social class I, non-smokers, high adherence to the Mediterranean diet, physically active, and normal social support.

## Discussion

### Main findings

In this large occupational cohort of Spanish workers, we found that obesity indices—BMI, WtHR, CUN-BAE, and METS-VF—were strongly associated with sociodemographic characteristics, lifestyle behaviors, and perceived social isolation. Male sex, older age, lower social class, physical inactivity, poor adherence to the Mediterranean diet, smoking, and higher levels of social isolation were consistently linked to greater odds of obesity, irrespective of the index applied. Sensitivity analyses excluding participants with extreme BMI values (<18.5 or >40 kg/m²) and those with missing covariates yielded results consistent with the main findings. These analyses confirmed the robustness of the associations and are presented in **Supplementary Tables S5**, **S6**.

### Comparison with previous studies

Our findings are consistent with prior epidemiological research that has established sociodemographic disparities in obesity prevalence. Multiple studies have shown that men and older adults exhibit higher rates of central and visceral obesity, measured by WtHR or METS-VF, compared to women and younger populations (50, 51). Lower socioeconomic position is also a recognized determinant of obesity, partly due to disparities in access to healthy foods, occupational demands, and opportunities for physical activity (52, 53). With respect to lifestyle habits, poor diet quality and low levels of physical activity have been repeatedly associated with increased obesity risk in European and Mediterranean cohorts (54, 55). The Mediterranean diet, in particular, has been inversely related to obesity and central adiposity, reflecting its emphasis on nutrient-rich, anti-inflammatory foods (56).

Our study also reinforces emerging evidence linking social isolation to obesity. Recent population-based research has identified social isolation and poor social support as independent predictors of obesity and related cardiometabolic outcomes (42, 57). The mechanisms through which social isolation may contribute to obesity are multifaceted. Isolation due to illness may reduce opportunities for physical activity, while isolation associated with aging or lack of social support can increase sedentary behavior and unhealthy dietary patterns (58, 59). These factors synergistically contribute to weight gain and adiposity, highlighting the importance of addressing psychosocial determinants in obesity prevention strategies (60). Mechanisms proposed include reduced engagement in healthy behaviors, higher prevalence of depression and stress-related eating, and altered physiological pathways involving the hypothalamic–pituitary–adrenal axis (61, 62). By integrating these findings into an occupational setting, our analysis highlights that psychosocial factors, alongside traditional determinants, play an important role in obesity development among working adults.

### Potential mechanisms

Several biological and behavioral mechanisms may explain the observed associations. Aging is accompanied by changes in body composition, including a decline in lean mass and preferential fat accumulation in visceral depots, which increases risk for metabolic dysfunction (63). Sex differences may relate to hormonal influences on fat distribution; premenopausal women typically store more subcutaneous fat, whereas men accumulate more visceral fat, which confers greater cardiometabolic risk (64). Lower social class may predispose individuals to energy-dense dietary patterns, reduced leisure time for physical activity, and higher occupational stress, all of which favor weight gain (65).

Lifestyle factors interact synergistically with these determinants. Physical inactivity reduces energy expenditure and alters mitochondrial function, while poor dietary quality promotes adiposity through excessive caloric intake and impaired satiety regulation (66). Smoking shows complex associations; while nicotine can reduce weight in the short term, smoking cessation is often followed by weight gain, and chronic smoking is linked to central obesity and metabolic dysfunction (67). Social isolation further exacerbates these pathways. Individuals with limited social networks may experience higher stress and reduced accountability for maintaining healthy routines, leading to maladaptive behaviors such as sedentary lifestyles and unhealthy eating (68). Physiologically, social isolation has been associated with dysregulation of cortisol, inflammatory cytokines, and autonomic balance, all of which can facilitate fat deposition and insulin resistance (69).

### Strengths and limitations

The strengths of this study include its very large sample size, the comprehensive evaluation of four obesity indices that capture distinct dimensions of adiposity, and the simultaneous consideration of sociodemographic, lifestyle, and psychosocial determinants. The inclusion of validated instruments for diet (MEDAS), physical activity (IPAQ), and social isolation (ESSI) enhances the reliability of the measurements. Furthermore, the stratified analyses by sex and the application of multiple sensitivity tests add robustness to the findings.

However, several limitations should be noted. First, the cross-sectional design prevents causal inference; associations may be bidirectional, particularly regarding social isolation and obesity. Longitudinal studies are necessary to clarify temporal relationships. Second, obesity indices, although validated, rely on anthropometric and bioelectrical estimations rather than gold-standard imaging techniques such as DXA or MRI. This may introduce misclassification, though the large sample size likely mitigates random error. Third, although adjustments were made for major confounders, residual confounding by unmeasured variables (e.g., genetic predisposition, occupational stressors, sleep quality) cannot be excluded. Fourth, social isolation was assessed with a brief validated instrument, which may not capture the full spectrum of social connectedness and loneliness. Finally, the study population consisted of Spanish workers, which may limit generalizability to unemployed individuals, older populations, or different cultural contexts.

Although sex was included as an adjusting variable in all multivariable models, preliminary stratified analyses showed consistent associations across men and women, without meaningful differences in direction or magnitude. For this reason, we did not present sex-stratified results, prioritizing clarity and conciseness in the main manuscript.

The consistency of the findings was further supported by sensitivity analyses (**Supplementary Tables S5**, **S6**), which excluded participants with extreme BMI values and those with missing covariates, showing that the associations remained robust across different model specifications.

### Implications for public health and future research

Our findings have important implications for public health and occupational health strategies. The consistent associations between obesity indices and sociodemographic and lifestyle determinants underscore the need for integrated interventions that go beyond individual-level counseling to address structural and social determinants of health. Workplace-based programs promoting adherence to the Mediterranean diet, physical activity, and smoking cessation may be particularly effective in reducing obesity burden. Additionally, the novel role of social isolation as a correlate of obesity highlights the importance of fostering social support and connectedness in preventive strategies. Interventions that integrate social engagement, peer support, and mental health promotion could yield dual benefits for obesity and overall well-being.

Future research should adopt longitudinal designs to unravel causal pathways linking social isolation and other psychosocial factors with obesity. Studies incorporating biomarkers of stress, inflammation, and neuroendocrine function could elucidate biological mediators. Moreover, comparisons across occupational groups and cultural settings will be necessary to determine the generalizability of these findings. Ultimately, multi-component interventions addressing sociodemographic disparities, lifestyle habits, and psychosocial well-being are likely to be most effective in tackling the obesity epidemic.

## Conclusions

In this large occupational cohort of Spanish workers, obesity indices—including BMI, WtHR, CUN-BAE, and METS-VF—were strongly associated with sociodemographic, lifestyle, and psychosocial determinants. Male sex, older age, lower social class, poor adherence to the Mediterranean diet, physical inactivity, smoking, and higher levels of social isolation emerged as consistent correlates of obesity across all indices. These findings underscore the multidimensional nature of obesity, reflecting the interplay of biological, behavioral, socioeconomic, and psychosocial factors.

The study contributes novel evidence by integrating social isolation into the framework of obesity determinants, demonstrating that individuals with limited social support are at higher risk of excess adiposity. This highlights the importance of considering psychosocial environments in addition to traditional biomedical and behavioral factors when addressing obesity.

From a public health perspective, the results emphasize the need for comprehensive prevention strategies that simultaneously target lifestyle behaviors, reduce socioeconomic disparities, and enhance social connectedness. Workplace-based interventions may represent an effective avenue to promote healthy eating, physical activity, and supportive social environments, particularly in populations at elevated cardiometabolic risk.

Future research should prioritize longitudinal studies to clarify causal pathways, assess the role of psychosocial stress and biological mediators, and explore the generalizability of these findings across cultural and occupational settings. Ultimately, tackling obesity will require a multi-level approach that addresses not only individual behaviors but also the broader social and structural determinants of health.

## Data Availability

The raw data supporting the conclusions of this article will be made available by the authors, without undue reservation.
