# Supplementary material for: Obesity indices and their sociodemographic, lifestyle, and social isolation correlates in a large Spanish working population
Source: Front Endocrinol (Lausanne). 2025 Oct 10;16:1695705. doi: 10.3389/fendo.2025.1695705 (PMC12549272; doi:10.3389/fendo.2025.1695705)
Supplement: Supplementary file 1 [file Table1.docx]

# Supplementary Material

## Supplementary Table S1

Sensitivity analysis excluding participants with extreme BMI values (<18.5 or >40 kg/m²). Multivariable logistic regression models assessing the association between sociodemographic, lifestyle, and social isolation variables with obesity indices.

| Variable | OR (95% CI) | p-value |
| --- | --- | --- |
| Age (per 10 years) | 1.25 (1.18–1.32) | <0.001 |
| Male sex | 1.40 (1.28–1.52) | <0.001 |
| Low educational level | 1.18 (1.10–1.27) | <0.001 |
| Manual occupation | 1.15 (1.07–1.24) | <0.001 |
| Current smoking | 0.95 (0.89–1.02) | 0.12 |
| Low physical activity | 1.30 (1.21–1.39) | <0.001 |
| Poor adherence to Mediterranean diet | 1.22 (1.14–1.30) | <0.001 |
| Social isolation (high) | 1.35 (1.22–1.49) | <0.001 |

Note: Results remain consistent with the main analysis, indicating robustness to the exclusion of participants with extreme BMI values.

## Supplementary Table S2

Sensitivity analysis excluding participants with missing covariates. Multivariable logistic regression models assessing the association between sociodemographic, lifestyle, and social isolation variables with obesity indices.

| Variable | OR (95% CI) | p-value |
| --- | --- | --- |
| Age (per 10 years) | 1.23 (1.17–1.30) | <0.001 |
| Male sex | 1.42 (1.30–1.55) | <0.001 |
| Low educational level | 1.16 (1.08–1.25) | <0.001 |
| Manual occupation | 1.12 (1.04–1.21) | 0.002 |
| Current smoking | 0.97 (0.91–1.04) | 0.36 |
| Low physical activity | 1.28 (1.19–1.37) | <0.001 |
| Poor adherence to Mediterranean diet | 1.20 (1.12–1.28) | <0.001 |
| Social isolation (high) | 1.33 (1.20–1.46) | <0.001 |

Note: The associations were robust across sensitivity analyses, supporting the multifactorial determinants of obesity.
